# Coordination Engineering of Ultra‐Uniform Ruthenium Nanoclusters as Efficient Multifunctional Catalysts for Zinc–Air Batteries

**DOI:** 10.1002/smsc.202200035

**Published:** 2022-08-11

**Authors:** Yingying Guo, Donghai Wu, Minhan Li, Kaixi Wang, Shouren Zhang, Guangli He, Hengbo Yin, Chenyu Huang, Baocheng Yang, Jianan Zhang

**Affiliations:** ^1^ Henan Provincial Key Laboratory of Nanocomposite and Applications Institute of Nanostructured Functional Materials Huanghe Science and Technology College Zhengzhou Henan 450006 P. R. China; ^2^ College of Materials Science and Engineering Zhengzhou University Zhengzhou 450001 P. R. China

**Keywords:** hydrogen evolution reaction, N,S co-doped carbon, Ru nanoclusters, S-C sites, zinc-air batteries

## Abstract

The lack of highly efficient, inexpensive catalysts severely hinders the large‐scale application of electrochemical energy conversion technologies (e.g., electrochemical hydrogen evolution reaction (HER) for hydrogen production, metal–air batteries (Cathode: oxygen reduction reaction (ORR))). As a new class of nanomaterials with a high ratio of surface atoms and tunable composition and electronic structure, metal nanocluster (NCs) are promising candidates as catalysts. Herein, a novel catalyst using S,N‐doped carbon matrix (NSCSs) is synthesized to efficiently stabilize high density and ultra‐uniform ruthenium (Ru) nanoclusters (Ru@NSCSs) by small‐molecule self‐assembly pyrolysis approach. The obtained Ru@NSCSs catalyst exhibits outstanding HER activity in all pH conditions (especially with a low overpotential of 5 mV at a current density of 10 mA cm^−2^ in 1 m KOH) and excellent ORR performance (half‐wave potential (*E*_1/2_) of 0.854 V in 0.1 m KOH). Based on the experimental investigations and theoretical calculations, it is discovered that the S‐atom can modulate the electronic structure and optimization of redox states on the surficial sites of Ru NCs during the ORR process. This work provides a feasible strategy for understanding and regulating the metal–support interface of ultra‐uniform nanoclusters catalysts.

## Graphical abstract

A novel catalyst using S, N‐doped carbon matrix is synthesized to efficiently stabilize high density and ultra‐uniform ruthenium (Ru) nanoclusters (Ru@NSCSs) by small‐molecule self‐assembly pyrolysis approach. The centerpiece is the coordination of N, S atoms that optimizes the interface structure of *Ru_S,N_ sites, which is beneficial to the development of multifunctional and highly active catalysts.

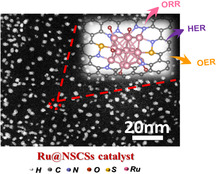

## 1 Introduction

Electrocatalysis is the core foundation of electrochemical energy conversion and storage, such as water electrolysis systems, metal–air batteries, fuel cells, which have become current research hotspots.^[^ 1, 2, 3, 4, 5 ^]^ Currently, the most widely used reaction catalyst is a Pt‐based catalyst, but it is expensive and resource scarce. Ru‐based catalysts, as a promising alternative to Pt, have drawn great attention due to their relatively low cost and outstanding activities in hydrogen evolution reaction (HER), OER, and oxygen reduction reaction (ORR).^[^ 6, 7, 8, 9, 10, 11, 12, 13 ^]^ To balance the relationship between cost and catalytic activity, a growing number of ultra‐small size metal nanoclusters (NCs) and single atoms have been elaborately prepared.^[^ 14, 15, 16, 17 ^]^ Stemming from the unique geometric and electronic structures, supported metal NCs can provide more active sites compared to single atoms, and the orbital overlap between atoms increases the opportunity to manipulate electronic structures,^[^ 14, 18, 19 ^]^ which is beneficial to the development of multifunctional and highly active catalysts. But, due to the strong interaction between the complexing group of substrates and the metal ion, the NCs with high‐density dispersion, well crystallinity, and high‐temperature resistance is very difficult to obtain as a model for in‐depth investigation of their physicochemical and catalytic properties. Some emerging synthetic strategies of supported atomic NCs have been reported, such as atomic‐layer deposition,^[^ ^20^ ^]^ wet chemistry reduction,^[^ ^21^ ^]^ precursor‐preselected strategy,^[^ ^22^ ^]^ etc. However, when the particle size is lower than 2 nm, the thermodynamics is unstable. The essential reason is the loss of its unique structure and properties caused by particle migration and coalescence (PMC) and/or Ostwald ripening (OR) processes at high temperature.^[^ 23, 24 ^]^ Therefore, it is urgent to develop effective methods to stabilize the ultra‐small size (≤2 nm) and high dispersion NCs on carbon substrate up to 700 °C.

The heteroatom‐doped carbon materials have excellent conductivity, high surface area, and abundant defects, which are conducive to the distribution and stability of the atomic NCs via ligand protection from heteroatom coordination.^[^ ^25^ ^]^ For example, carbon nitride with abundant N atoms can be employed to coordinate and stabilized the Fe_2_ NCs.^[^ ^14^ ^]^ Liu et al encapsulated Ir precursor into the S‐doped carbon, followed by pyrolysis for forming Ir_13_ NCs, where strong metal–support interactions between the Ir atoms and the carbon‐based carrier effectively stabilize and limit the agglomeration of Ir atoms.^[^ ^26^ ^]^ In another example, Liang et al. reported the S‐doped carbon matrix can efficiently stabilize ≈1 nm metal NCs (Pt, Ru, Rh, Os, and Ir) against thermal sintering at high temperatures up to 700 °C in a reductive atmosphere.^[^ ^24^ ^]^ Consequently, the supports can provide the anchoring sites to hinder the migration and aggregation sintering mechanism of metals at high temperatures. The stabilization effect of coordination heteroatoms on defective carbon carriers holds promising to preserve the high dispersion of sub‐nanometric metal species under harsh reaction conditions, especially at high temperatures (≥700 °C). Therefore, investigating the role of heteroatom‐doped carbon defect mechanisms in the thermal decomposition and synthesis of sub‐nano‐NCs is imperative for achieving controllable preparation of carbon‐based nanoclusters with low cost and high catalytic performance.

Herein, a facile strategy has been provided for synthesizing ultra‐uniform Ru NCs dispersed on S,N‐doped carbon matrix (NSCSs) , through direct pyrolysis of a mixture of N‐acetyl‐L‐cysteine, ruthenium (III) chloride hydrate (RuCl_3_·*x*H_2_O), and melamine in an argon atmosphere at 900 °C. The catalyst shows excellent HER activity with small overpotentials at 10 mA cm^−2^ (5 mV in 1.0 m KOH, 89 mV in 0.5 m H_2_SO_4_, and 98 mV in 1.0 m PBS, vs RHE). Importantly, the obtained catalyst exhibits good stability with a negligible current decrease after continuous testing for 10 h under all pH conditions. Furthermore, when the Ru@NSCSs are used as an ORR cathode catalyst, it shows better ORR activity and stability. According to electrochemical test results and X‐ray absorption fine structure (XAFS) analysis, we conclude that the catalyst with Ru–S/Ru–N active center can enable Ru NCs to be resistant against sintering up to 900 °C. The density function theory (DFT) calculations demonstrate that the electronic interaction between the Ru NCs and the S‐atoms could tune the adsorption strength of *O and *OH on Ru NCs sites, so *Ru_S,N_ sites have high activity for ORR. Moreover, when assembled into a liquid zinc–air battery, this catalyst displays a superior peak power density of 151 mW cm^−2^ and has long‐term cycle durability (100 h at 5 mA cm^−2^) in liquid and reversible flexible all‐solid‐state battery.

## 2 Results and Discussion

**Figure 1:**
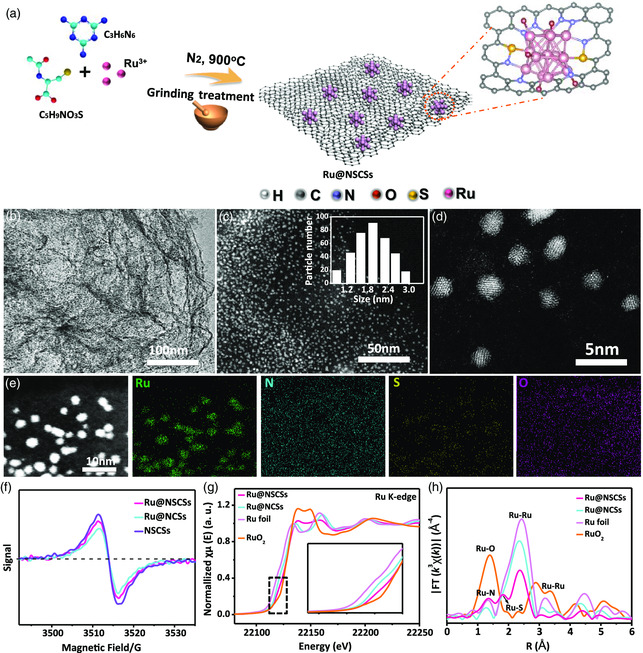
a) Synthetic procedure of the Ru nanoclusters (NCs) embedded in N, S co‐doped defect carbon nanosheets (Ru@NSCSs). b) Transmission electron microscopy (TEM) images of Ru@NSCSs. c–d) high‐angle annular dark‐field‐TEM (HAADF‐TEM) images (Inset in c: corresponding particle size distribution of Ru NCs based on a count of 360 in the sample area), scale bar: 20 nm. e) Dark‐field scanning TEM (STEM) image and energy‐dispersive X‐ray (EDX) elemental mappings, scale bars: 10 nm. f) EPR spectra. g) The normalized Ru Kedge X‐ray absorption near edge structure (XANES) spectra and h) The K^3^‐weighted Ru k‐edge Fourier transform EXAFS spectra of Ru@NSCSs, Ru@NCSs and reference Ru foil and RuO_2_ samples.

**Figure** **1**a illustrates the synthesis process of the Ru@NSCSs catalyst using S, N‐doped carbon matrix to efficiently stabilize high density and ultra‐uniform ruthenium (Ru) nanoclusters by a small‐molecule self‐assembly pyrolysis approach. 20 mg of RuCl_3_·*x*H_2_O, 150 mg of N‐acetyl‐L‐cysteine, and melamine were added to the ethanol solution. The obtained mixture was stirred for 60 min and dried at 80 °C overnight. Then, the small molecules in the precursor self‐assembled to form Ru nanoclusters with N, S heteroatoms co‐anchored in a high‐temperature heat treatment at 900 °C. By removing N‐acetyl‐cysteine in the precursor slurry and keeping the other conditions unchanged, the S‐free N‐doped carbon (Ru@NCSs) could be made. Additionally, we synthesized Ru single‐atom catalysts to demonstrate the higher activity of Ru nanoclusters. To further verify the presence of Ru NCs, XRD patterns were performed for the Ru@NSCSs, Ru_SA_@NSCSs, Ru@NCSs, and NSCSs, and the obtained results are shown in Figure S1, Supporting Information. Obviously, the Ru NCs were successfully anchored in the carbon skeleton by the appearance of typical carbon peaks at 26.2° for (002) facet and 44.3° for (101) facet, and weak peaks at 40.7° and 47.4°can be well indexed to the (111) and (200) planes of Ru@NSCSs and Ru@NCSs, respectively.^[^ ^27^ ^]^ From the XRD in Ru_SA_@NSCSs and NSCSs, only a broad at 25° can be observed (Figure S1, Supporting Information), which corresponds to the interlayer distance of the carbon matrix. From the scanning electron microscopy (SEM, Figure S2, Supporting Information) and transmission electron microscope (TEM, Figure S3, Supporting Information) images, we know that the as‐prepared NSCSs and Ru_SA_@NSCSs show a nanosheet structure, and no nanoparticles or clusters were found in the high‐resolution image of Ru_SA_@NSCSs. This further proves that we successfully prepared Ru_SA_@NSCSs catalyst. Aberration‐corrected high‐angle annular dark‐field scanning TEM (HAADF‐STEM) measurements of the catalyst also confirm that the distribution of Ru NCs with size in the narrow range of 1.6–2.2 nm (mean size, 2.0 nm) are embedded into carbon matrix, without any large particles or aggregates (Figure 1b–d and S4, Supporting Information). Surprisingly, as shown in the HAADF‐STEM and TEM images (Figure S5, Supporting Information), Ru@NCSs show obvious agglomeration. The catalyst clearly shows some aggregation and broad particle size distribution ranging from a few to around ten nanometers on the whole carbon matrix. Through analysis, we can conclude that these S atoms are conducive to hindering the diffusion of metal atoms and avoiding the agglomeration of NCs after annealing at 900 °C.

The fast Fourier transform (FFT) pattern and TEM images of Ru@NSCSs show that Ru NCs embedded in graphitic carbon and lattice fringes with a spacing of 0.223 and 0.192 nm, which are attributed to the (111) and (200) crystalline planes of metallic face‐centered cubic (FCC) structure, respectively (Figure S6, Supporting Information). The formation of the Ru@NSCSs catalyst is confirmed by energy‐dispersive X‐ray (EDX) analysis in HAADF‐STEM manifests homogeneous distribution of C, O, N, S, and Ru elements (Figure 1e and S7, Supporting Information). The content of Ru@NSCSs was determined by thermogravimetric analysis (TGA) in air, and its weight was about 33.27 wt% (Figure S8, Supporting Information). In the TGA test, the remaining weight is supposed to be the weight of RuO_2_. From the results of inductively coupled plasma (ICP) analysis, the loading of Ru in Ru@NSCSs and Ru@NCSs was 2.66 wt% and 2.72 wt% (Table S1, Supporting Information), respectively. The as‐prepared Ru@NSCSs have a large Brunauer–Emmett–Teller (BET) surface area of 414.576 m^2^ g^−1^ and a pore volume of 0.86 cm^3^ g^−1^ (Figure S9a and Table S2, Supporting Information), and the hierarchical pore sizes of 3.8 and 32.1 nm (Figure S9b, Supporting Information). It helps to disperse Ru NCs and provides a good space for the anchoring of Ru NCs.^[^ ^28^ ^]^ Moreover, from the recorded Raman spectra shown in Figure S10, Supporting Information, we can observe two peaks at 1347 and 1598 cm^−1^, which can be ascribed to the D‐band and G‐band of carbon. The *I*_D_/*I*_G_ ratio of Ru@NSCSs is greater than that of Ru@NCSs, signifying that the introduction of S‐atom brings more structural defects. Moreover, the analysis of the results according to the electron paramagnetic resonance (EPR) spectrum also shows that Ru@NSCSs have more inherent defects than Ru@NCSs (Figure 1f).^[^ 29, 30 ^]^

To gain insight into the chemical compositions in samples and their corresponding electronic states, X‐ray photoelectron spectroscopy (XPS) was performed. The XPS spectra of Ru@NSCSs show distinct signals of S, C, N, O, and Ru elements (Figure S11 and Table S3, Supporting Information). The peaks of the NSCSs sample at about 284.6, 285.5, and 288.0 eV in the C 1*s* spectrum belong to the C=C bond, C=N bond, and C—N bond (Figure S12a, Supporting Information). Figure S12b–c, Supporting Information, shows the C 1*s* + Ru 3*d* XPS spectra of the Ru@NSCSs and Ru@NCSs catalyst. Ru 3*d* XPS spectra of Ru@NSCSs and Ru@NCSs samples with a Ru 3*d*_3/2_ peak at 284.6 eV and a Ru 3*d*_5/2_ peak at 280.3 eV,^[^ 16, 31 ^]^ and the peak with energy 280.3 eV can be attributed to metallic Ru (0), indicating that Ru NCs were successfully incorporated into NSCSs. Meanwhile, we can also see C=C (284.6 eV), C=N (285.5 eV), and C—N (288.0 eV) peaks on the binding energies of Ru@NSCSs and Ru@NCSs, while the peaks around 280.3 eV belong to zero‐valent Ru. The high‐resolution N 1*s* spectrum of NSCSs in Figure S13a, Supporting Information, could be deconvoluted into four peaks of oxidized‐N at 403.7 eV, graphitic‐N at 400.8 eV, pyridinic‐N at 399.1 eV, and pyrrolic‐N at 397.8 eV. When Ru NCs were introduced, it was concluded by analyzing the XPS results of Ru@NSCSs and Ru@NCSs in Figure S13b,c, Supporting Information, that the oxidation‐N, graphitic‐N, pyridinic‐N, and pyrrolic‐N have more Ru–N peaks at 398.4 eV, indicating that Ru NCs coordinate with N species in Ru@NSCSs and Ru@NCSs. Figure S14a, Supporting Information, shows the O 1*s* peak of the NSCSs catalyst, the main two peaks can be contributed to the C=O bond at 532.7 eV, C—O—C/C—OH bond at 531.2 eV, respectively. The O 1*s* spectra of Ru@NSCSs and Ru@NSCSs in Figure S14b,c, Supporting Information, the catalysts can be deconvoluted into four peaks with their binding energies at 530.2, 531.2, and 532.72 eV, assigned to the lattice oxygen of Ru‐O, hydroxyl groups adsorbed on the surface of Ru (Ru–OH) and oxygen bound with carbon (C=O), respectively.^[^ 32, 33 ^]^ The aforementioned results indicate that Ru NCs are most possibly bonded to O at high temperatures. For the S 2*p* spectra in the NSCSs samples, peaks were observed around 168.8, 164.8, and 163.7 eV, corresponding to the oxidized S, S 2*p*_1/2,_ and S 2*p*_3/2_ splitting of the S 2*p* spin orbitals (C–S–C), respectively (Figure S15a, Supporting Information). Meanwhile, Ru@NSCSs also exhibited similar peaks in binding energy (Figure S15b, Supporting Information). Surprisingly, another peak around 162.8 eV was observed in Ru@NSCSs. According to previous literature reports, we know that this peak can be attributed to the M—S bond, so it corresponds to the Ru—S bond.^[^ 34, 35 ^]^ Then, X‐ray absorption near edge structure (XANES) and extended X‐Ray absorption fine structure (EXAFS) were performed to verify the atomic structure of the samples. As shown in Figure 1g, the near‐edge absorption energy and the white line intensities of Ru@NSCSs and Ru@NCSs are located between Ru foil and closer to the RuO_2_ sample, this confirms that the oxidation state of the Ru atoms was below +4.^[^ 34, 36 ^]^ In the normalized Fourier transform of the extended X‐ray absorption fine structure (FT‐EXAFS) curves in Figure 1h and S16, Supporting Information, the Ru@NSCSs have a main peak around 1.36 Å, which is attributed to the Ru–N coordination, and the contribution of Ru–S coordination is about 1.8 Å. This result supports the coexistence of Ru NCs and Ru–S/Ru‐N sites in the Ru@NSCSs catalyst. Differently, the Ru@NCSs catalyst shows the Ru–N coordination at ≈1.36 Å, without the Ru–S coordination environment.^[^ ^34^ ^]^ In addition, there is a distinct Ru–Ru peak in the Ru@NSCSs catalyst, which is related to the Ru–Ru interaction, and simultaneously appears in Ru@NSCSs, Ru@NCSs, and metallic Ru. Note that the Ru–Ru peak intensity of the Ru@NCSs is higher than Ru@NSCSs, owing to the existence of Ru NCs with broad size distribution in Ru@NCSs. Thus, Ru nanoclusters exist stably and uniformly through the coordination interaction of N, S, and O atoms on the defective carbon nanosheets.

**Figure 2:**
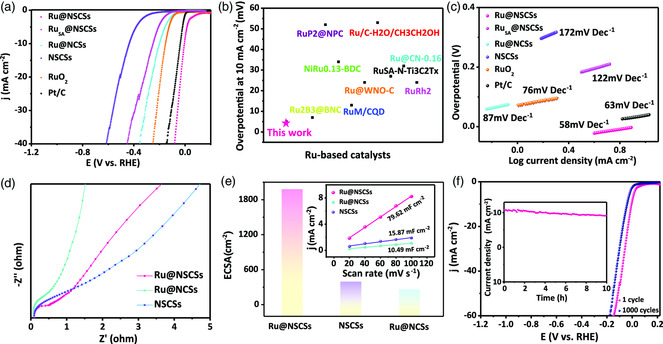
a) Hydrogen evolution reaction (HER) polarization curves of Ru@NSCSs, Ru_SA_@NSCSs, Ru@NCSs, NSCSs, RuO_2,_ and commercial Pt/C catalyst at a sweep rate of 5 mV s^−1^ in 1 m KOH solution. b) Activity comparison of Ru@NSCSs with other recently reported Ru‐based catalysts in the overpotential at 10 mA cm^−2^ (seen Table S4, Supporting Information for more details for the comparison). c) Correspond to the Tafel plots for these catalysts. d) Nyquist plots of Ru@NSCSs, Ru@NCSs, and NSCSs in 1 m KOH solution. e) Electrochemical surface area (ECSA) values of Ru@NSCSs, Ru@NCSs, and NSCSs catalysts. Inset of estimation of *C*_dl_ by plotting the half of current density variation (Δ*j* = *j*_anodic_−*j*_cathodic_). ECSA values at the overpotentials of 200 mV. f) The polarization curves were recorded initially and after 1000 cycles. Inset shows the chronopotentiometry of Ru@NSCSs catalysts.

The interaction between the coordination atoms (N, S, and O) and Ru NCs may cause synergistic effects for catalytic application. Inspired by the Ru‐based catalysts with excellent HER performance in a wide pH range, we further studied the intrinsic electrocatalytic performances of prepared samples in the HER reaction. The HER polarization curves of Ru@NSCSs, Ru_SA_@NSCSs, Ru@NCSs, NSCSs, RuO_2,_ and commercial Pt/C catalyst were tested in 1 m KOH, 0.5 m H_2_SO_4,_ and 1 m PBS solution at a sweep rate of 5 mV s^−1^, respectively.^[^ ^37^ ^]^ Remarkably, the Ru@NSCSs displays high HER activity with a low overpotential of 5 mV at 10 mA cm^−2^ in 1 m KOH solution, which is much lower than that of Ru_SA_@NSCSs (*η* = 249 mV), Ru@NCSs (*η* = 198 mV) and NSCSs (*η* = 435 mV) (**Figure** **2**a, and Table S4, Supporting Information). Compared with Ru_SA_@NSCSs, Ru@NSCSs have multiple coupling active sites, which is conducive to promoting the kinetics of hydrogen evolution. Also, the Ru@NSCSs exhibit the best HER activity compared with the Pt/C (*η* = 38 mV) and RuO_2_ (*η* = 152 mV) at the overpotential at 10 mA cm^−2^ (Figure 2b, and Table S4, Supporting Information), making it be one of the best HER catalysts to recently reported Ru‐based electrocatalysts. After that, we further tested the oxygen evolution reaction (OER) performance of the catalysts under alkaline conditions. The as‐prepared Ru@NSCSs exhibited good OER activity in 1 m KOH solution at a voltage of 1.54 V and a current of 10 mA cm^−2^. Meanwhile, Ru@NSCSs decayed by 30 mV after 1000 cycles at a scan rate of 100 mV s^−1^ **(**Figure S17, Supporting Information). The electrocatalytic performance was also tested in 0.5 m H_2_SO_4_ and 1 m PBS electrolyte **(**Figure S18a,d, Supporting Information), the Ru@NSCSs also exhibit the best HER activity among these catalysts, the overpotentials that deliver a current density of 10 mA cm^−2^ in 0.5 m H_2_SO_4_ and 1.0 m PBS were 89 and 98 mV, respectively. To investigate the intrinsic reaction kinetics of Ru@NSCSs for HER, the Tafel slope was plotted and fitted, the catalyst displays a smaller Tafel slope of 58 mV dec^−1^, which is lower than that of Ru_SA_@NSCSs (122 mV dec^−1^), Ru@NCSs (87 mV dec^−1^), and NSCSs (172 mV dec^−1^) catalysts (Figure 2c). And, the Ru@NSCSs also present a small Tafel slope in 0.5 m H_2_SO_4_ and 1 m PBS electrolytes. Additionally, the Nyquist plots also suggest a small charge transfer resistance of Ru@NSCSs (Figure 2d), revealing the lower charge transfer resistance. These results demonstrate that Ru@NSCSs have faster HER kinetics than Ru@NCSs and NSCSs, confirming the significant influence of S doping on increasing HER activity. The aforementioned merits of the Ru@NSCSs, including low overpotential and Tafel slope, are superior to most previously reported catalysts in the acidic, alkaline, and neutral solutions (Table S5, Supporting Information).

We further estimated the relative surface area of the samples by electrochemical cyclic voltammetry (CV) and studied the intrinsic activity of the samples. Typically, we evaluated the electrochemical surface areas (ECSA) of different catalysts based on electrochemical double layer capacitance (*C*_dl_) measurement against different scan rates (Figure S19–S21, Supporting Information).^[^ ^38^ ^]^ As seen from Figure 2e inset, with the addition of the S element, the *C*_dl_ proportional to ECSA increases from 0.15 mF cm^−2^ for Ru@NCSs to 9.93 mF cm^−2^ for Ru@NSCSs. This suggests that the Ru@NSCSs have the greatest number of electrochemical surface areas for HER in 1 m KOH solution. Meanwhile, we also prove that the Ru@NSCSs catalyst shows a high active area in 0.5 m H_2_SO_4_ and 1 m PBS, respectively. (Figure S17b,e, Supporting Information). Furthermore, we evaluated the stability by voltammetry sweeps and chronoamperometry tests of the samples in 1 m KOH, 0.5 m H_2_SO_4_, and 1 m PBS solution, respectively. After 1000 cycles, there is some slight dip (Figure 2f). We also explored the catalyst after the chronoamperometry tests (inset of Figure 2f), which shows the neglectable decay at 10 mA cm^−2^ for 10 h. According to the HER reaction mechanism, the attenuation mechanism is mainly due to the extraction of a large number of active sites of Ru metal, resulting in the deformation of the optimal coordination, resulting in the attenuation of the HER performance. Then, we also tested the stability of the catalyst under acidic and neutral conditions, the Ru@NSCSs exhibits good stability with a negligible current decrease after 10 h of the test (Figure S18c,f, Supporting Information). Overall, the Ru@NSCSs possess outstanding intrinsic activity and stability in HER, which mainly originates from the Ru–S/Ru–N sites to enhance the electronic structure of Ru NCs coupled with carbon support.

**Figure 3:**
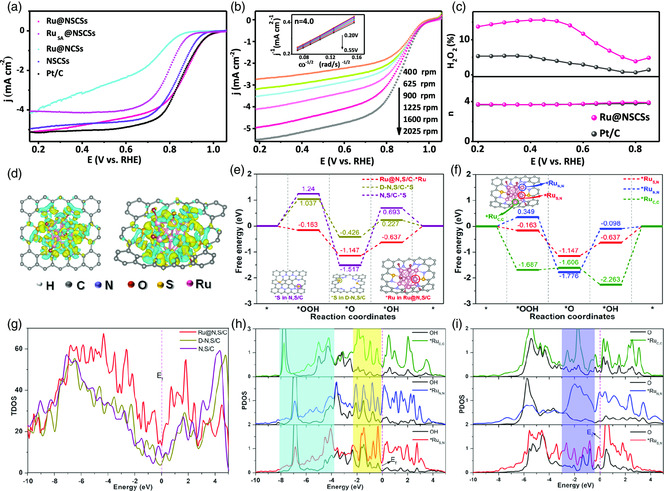
a) Linear sweep voltammetry (LSV) curves of Ru@NSCSs, Ru_SA_@NSCSs, Ru@NCSs, NSCSs, and commercial Pt/C catalyst in O_2_‐saturated 0.1 m KOH solution. b) LSV curves at different rotation rates in rpm. Inset is the corresponding *K‐L* plot with a sweep rate of 5 mV s^−1^. c) H_2_O_2_ yield and the electron transfer (*n*) in 0.1 m KOH solution. DFT calculation. d) The isosurface of the charge density difference for Ru13@N, S/C. On the left is the top view, and on the right is the side view. The C atoms are in gray, N in blue, S in yellow and O in red, H in silver, and Ru in pink, respectively. e) DFT study of free energy diagram for oxygen reduction reaction (ORR) at *U*_RHE_ = 1.23 V on S in N, S/C, S in D‐N, S/C and Ru in Ru@N, S/C sites, the inserted model is the intermediate structures of OOH*, O* and OH*. f) DFT study of free energy diagram for ORR on Ru_S,N_, Ru_N,N_ and Ru_C,C_ sites. The projected density of states (PDOS) distributions for g) Ru@N, S/C, D‐N, S/C and N, S/C. h) OH* an i) O* for Ru_S,N_, Ru_N,N_ and Ru_C,C_ sites with the aligned Femi level.

The strong metal–support interaction can be employed to encapsulate and confine the Ru NCs, the abundant N, S sites are favorable for optimizing the support structures and enhancing the intrinsic activity of Ru NCs. After that, we further explored the ORR activity and stability of Ru@NSCSs catalyst.^[^ 39, 40 ^]^ As shown in **Figure** **3**a and Table S6, Supporting Information, the linear sweep voltammetry (LSV) curves results illustrate that the Ru@NSCSs exhibit good ORR activity with an onset potential (*E*_onset_) of 1.01 V (vs RHE), half‐wave potential (*E*_1/2_) of 0.854 V and kinetic current density (*J*_k_) of −5.11 mA cm^−2^. In contrast, the catalytic performance of Ru_SA_@NSCSs (*E*_1/2_ = 0.79 V and *J*_k_ = 4.12 mA cm^−2^), Ru@NCSs (*E*_1/2_ = 0.652 V and *J*_k_ = 4.178 mA cm^−2^) and NSCSs (*E*_1/2_ = 0.831 V and *J*_k_ = 4.946 mA cm^−2^) are apparently inferior to that of Ru@NSCSs, which further suggests the S atoms can further improve Ru NCs catalytic performance. Importantly, the high *J*_k_ value of Ru@NSCSs is a benefit to the high power densities of metal‐air batteries. As for reaction kinetics, the Ru@NSCSs are confirmed by the lower Tafel slope (64 mV dec^−1^) compared to Pt/C (69 mV dec^−1^) (Figure S22, Supporting Information). The Koutecky–Levich (K–L) plots in Figure 3b and the inset of Figure 3b, the electron transferred number (*n*) of about 4.0 over the potential range from 0.2 to 0.45 V (vs RHE). Furthermore, the *n* is 4.0 and the H_2_O_2_ yield remains below 15% over the potential range of 0.2–0.9 V, elucidating the efficient four‐electron transfer process and high ORR selectivity (Figure 3c). When introducing 3 M methanol into 0.1 m KOH solution, the relative current density of Ru@NSCSs remained at 75%, while Pt/C showed a significant current decrease fall to 61% in 0.1 m KOH solution (Figure S23a, Supporting Information). The sample an excellent ORR catalyst with a high selectivity to the methanol crossover effect. To further verify the ORR activity and stability, we obtained the limiting current by chronoamperometry measurements of Ru@NSCSs catalyst decrease 26% after the 40 000 second run, while Pt/C showed a faster current loss in 15 000 s (Figure S23b, Supporting Information). Besides, after 5000 cycles of CV scanning in 0.1 m KOH solution, the *E*_1/2_ decay of Ru@NSCSs is 24 mV (Figure S23c,d, Supporting Information), indicating that its durability is superior to that of Ru@NCSs catalysts.

To elucidate the pivotal role of the cooperative mechanism between Ru NCs and N, S atoms, and the origin of high catalytic activity toward ORR, we carried out the DFT calculations.^[^ 41, 42 ^]^ Combining experiments and characterization analysis, we adopt Ru_13_ NCs coordinated with both N and S atoms in the geometry as the model. For comparison, the S atom in N, S/C, and S atom in defect‐rich D‐N, S/C were also modeled for calculation. To reveal the electron redistribution behavior, the isosurface of the charge density difference analyses are shown in Figure 3d, which can be clearly seen that the electron around the Ru NCs center transfers to the adjacent carbonaceous substrate by the interfacial metal–support electronic interactions. Further, the Bader charge analysis also indicates the Ru atoms are positively charged in Ru@N,S/C with an average value of 0.65 eV, making the metal Ru center more positive and weakening the adsorption of OH* (Figure S24, S25, Supporting Information). The free energy diagram in Figure 3e shows that the first two steps of the ORR pathway of Ru@NSCSs under 1.23 V are downhill, indicating the exothermic and spontaneous nature. While the latter two steps need energy injection, and the Δ*G* value in the last elementary step (proton/electron transfer to *OH) is the largest, thus is the rate‐determining step for ORR on the samples.^[^ 43, 44 ^]^ On the active site of *Ru_S,N_ in Ru@N,S/C system, which possesses the lowest overpotential of 0.637 V. This fact indicates the coexistence of S‐defects and metal NCs plays a key role in accelerating the ORR reaction process. In addition, Figure 3f shows that the overpotentials at the *Ru_N,N_ (1.678 V) and *Ru_C,C_ (2.263 V) sites are much larger than those at the *Ru_S,N_ sites. These calculation results verify that the synergistic interaction between the Ru NCs and defect‐rich NSCSs has a remarkable effect on the reaction barrier of ORR, which in turn accelerates the catalytic process. Subsequently, the strong interaction between the Ru NCs and the NSCSs was evaluated by the total density of states near the Fermi level in Figure 3g. There is an apparent downshift of *Ru_C,C_ (cyan area in Figure 3h) and stronger overlaps between *OH and *Ru_C,C_ below the Fermi level (blue area in Figure 3h), implying the strongest adsorption of *OH on the *Ru_C,C_ among the three Ru active sites. In addition, there is stronger overlaps between *O and *Ru_N,N_ below the Fermi level (purple area in Figure 3i), indicating the strongest adsorption of *O on the *Ru_N,N_. According to the computation formula of Δ*G*_ORR_, neither too strong nor too weak adsorption of intermediate is conducive to the whole ORR process. Compared with *Ru_N,N_ and *Ru_C,C_ sites, the adsorption strength of *O and *OH on *Ru_S,N_ sites are moderate and supplement each other, so *Ru_S,N_ sites have the highest activity than *Ru_N,N_ and *Ru_C,C_ for electrocatalytic ORR. In brief, the strong metal‐support interaction can be employed to encapsulate and confine the Ru NCs. The abundant Ru‐S/Ru‐N sites are favorable for coordination, and the stability of NCs will remarkably enhance the catalytic activity for ORR on the catalyst.

**Figure 4:**
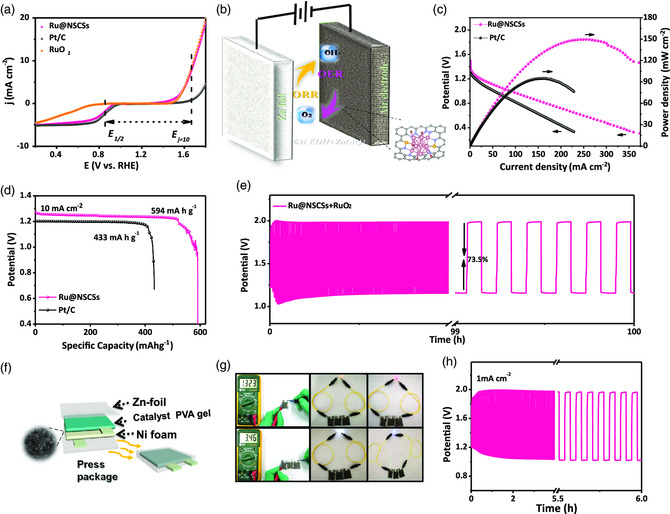
a) LSV curves of Ru@NSCSs catalysts in 0.1 m KOH, indicating the bifunctional activities toward both ORR and OER. b) Schematic representation of the rechargeable zinc–air battery. c) Polarization and power density curves of the Ru@NSCSs and Pt/C catalysts. d) Specific discharging capacities at 10 mA cm^−2^. e) Galvanostatic discharge–charge cycling curves of Ru@NSCSs at 5 mA cm^−2^. f) Schematic illustration and photographs of the all‐solid‐state zinc–air battery. g) Photograph of an all‐solid‐state zinc–air microbattery with an open‐circuit voltage of 1.323 V. Photograph of a lighted yellow, red, white, and blue LED (8 mm, ≈3.2 V) powered by three all‐solid‐state zinc‐air batteries interconnected in series. Open‐circuit voltage of ≈3.46 V. h) Discharge–charge cycling curves of flexible zinc–air battery.

The overall oxygen electrode activity can be evaluated by the difference between OER and ORR metrics. Obviously, the Ru@NSCSs exhibit superior ORR catalytic activity in alkaline electrolyte, for which we further tested the OER performance of the sample in 0.1 m KOH solution. The result implies that the as‐prepared Ru@NSCSs show remarkable intrinsic ORR and OER activities with the smallest Δ*E* (*E*_*j* = 10_–*E*_1/2_) value of 0.843 V in alkaline solution (**Figure** **4**a).^[^ ^45^ ^]^ Based on the aforementioned results, the Ru@NSCSs catalyst is assembled into the cathode electrode of a liquid zinc–air battery (Figure 4b).^[^ 46, 47 ^]^ Figure 4c shows the polarization and corresponding peak power density of 151 mW cm^−2^, better than that of commercial Pt/C (96 mW cm^−2^). The Ru@NSCSs have a specific capacity of 594 mAh g^−1^ at 10 mA cm^−2^, superior to commercial Pt/C (433 mAh g^−1^) (Figure 4d). The zinc–air battery produces an initial charge potential of 1.99 V and discharge potential of 1.156 V, with a small voltage gap of 0.834 V and a high round‐trip efficiency of ≈73.5%, very low charge–discharge polarization, and excellent reversibility of 100 h at 5 mA cm^−2^ at the scan rate of 600 s per cycle. According to the galvanostatic discharge–charge cycling curve at 5 mA cm^−2^ for 100 h, the sample shows excellent cycling stability (Figure 4e). Besides, we assembled flexible all‐solid‐state zinc–air batteries for the practical application of the prepared catalyst (Figure 4f).^[^ 48, 49, 50 ^]^ The device with Ru@NSCSs displays a desirable open‐circuit voltage (OCV) of around 1.323 V. Three all‐solid‐state zinc‐air batteries interconnected in series can easily light up the yellow, red, white, and blue LEDs with a voltage of about 3.46 V (Figure 4g). Afterward, we further tested the cycling stability of the assembled solid‐state zinc–air battery for 5 h of discharge–charge at 1 mA cm^−2^ (Figure 4h). In general, Ru@NSCSs has shown good prospects in the development of high‐performance zinc–air batteries.

## 3 Conclusions

In summary, we designed and synthesized the uniform dispersion and ultra‐small Ru NCs anchoring in the carbon layer as effective HER, OER, and ORR catalysts. Based on the experimental and characterization, and theoretical calculations, we attribute the crucial role of the defective S atoms in stabilizing metal NCs. When the Ru@NSCSs catalyst serves as cathode electrodes at all pH values, excellent catalytic stability was obtained, and the activity was further optimized. Moreover, as an ORR catalyst, Ru@NSCSs catalyst exhibits a half‐wave potential (*E*_1/2_) of 0.854 V in 0.1 m KOH. Remarkably, when it is applied to rechargeable liquid and flexible all‐solid‐state batteries, it has better cycle stability and ductility. The centerpiece is the coordination of N, S atoms that optimize the interface structure of *Ru_S,N_ sites, improves the adsorption strength of ORR intermediate products at the active center, reduces the reaction barrier of the decisive step OH* reduction, and improves the catalytic activity of ORR. This work not only develops a new path for achieving the ultra‐uniformly dispersed metal NCs, but also offers a brand‐new concept of interfacial engineering to guide the further design of multifunctional catalysts.

## 4 Experimental Section

4.1

4.1.1

##### Chemicals and Reagents

All chemicals were purchased and used without further purification. Ruthenium (III) chloride hydrate (RuCl_3_·*x*H_2_O), N‐Acetyl‐cysteine, melamine, and ethanol were obtained from Sinopharm Chemical Reagent Co., Ltd. Nafion (5.0 wt%) was purchased from Sigma‐Aldrich. All chemicals were used as received without any further purification. Pt catalyst (20% Pt supported on Vulcan XC‐72 carbon) was obtained from Johnson Matthey. Deionized water was used in all experiments.

##### Synthesis of Ru@NSCSs Catalyst

In a typical experiment, N‐acetyl‐cysteine (0.150 g), melamine (5 g), and RuCl_3_·*x*H_2_O (20 mg) were dissolved in 50 mL water under ultrasonic treatment at room temperature for 60 min, and then dried in an oven at 60 °C, and then pyrolyzed at 900 °C for 2 h under an N_2_ atmosphere and then was followed to cool naturally to room temperature, the contained black carbon material named Ru@NSCSs.

##### Synthesis of Ru_SA_@NSCSs Catalyst

By changing the content of RuCl_3_·*x*H_2_O to 0.1 mg and keeping the other conditions unchanged, the Ru_SA_@NSCSs could be made.

##### Synthesis of Ru@NCSs Catalyst

By removing N‐Acetyl‐cysteine in the precursor slurry and keeping the other conditions unchanged, the Ru@NCSs could be made.

##### Synthesis of NSCSs Catalyst

By removing RuCl_3_·*x*H_2_O in the precursor slurry and keeping the other conditions unchanged, the NSCSs could be made.

##### Characterizations

The morphology of the samples was studied by field‐emission (FE‐SEM, JEORJSM‐6700F) and transmission electron microscope (TEM, FEI Tecnai G2 20) with an accelerating voltage of 200 kV. Powder X‐ray diffraction (XRD) patterns were collected using a Y‐2000X‐ray Diffractometer using copper Kα radiation (*λ* = 1.5406 Å) at 40 kV, 40 mA. The N_2_ adsorption/desorption curve was determined by BET measurements using a Micromeritics ASAP 2020 surface area analyzer. The X‐ray photoelectron spectroscopy (XPS) measurements were performed with an ESCA LAB 250 spectrometer using a focused monochromatic Al Kα line (1486.6 eV) X‐Ray beam with a diameter of 200 μm. The X‐Ray absorption spectra (XAS) measurements for the Ru K‐edge were conducted in transmission mode (fluorescence mode) on beamline 12‐BM in the Advanced Photon Source at Argonne National Laboratory. XANES and EXAFS data reduction and analysis were analyzed by Athena and Artemis software.

## Conflict of Interest

The authors declare no conflict of interest.

## Supporting information

Supplementary Material

## Data Availability

The data that support the findings of this study are available from the corresponding author upon reasonable request.
