# Supplementary material for: Coordination Engineering of Ultra‐Uniform Ruthenium Nanoclusters as Efficient Multifunctional Catalysts for Zinc–Air Batteries
Source: Small Sci. 2022 Aug 11;2(10):2200035. doi: 10.1002/smsc.202200035 (PMC11935901; doi:10.1002/smsc.202200035)
Supplement: Supplementary file 1 — Supplementary Material [file SMSC-2-2200035-s001.pdf]

Supporting Information

Coordination engineering of ultra-uniform ruthenium nanoclusters as efficient multifunctional catalysts for zinc-air batteries

Yingying Guo, [a] Donghai Wu, [a] Minhan Li, [b] Kaixi Wang, [a] Shouren Zhang, *[,a] Guangli He, [a] Hengbo Yin, [b] Chenyu Huang, [a] Baocheng Yang, *[,a] and Jianan Zhang *[,b]

Y. Guo 1, D. Wu 1, M. Li 2, K. Wang 1, Prof. S. Zhang 1, G. He 1, H. Yin 2, C. Huang 1, Prof. B. Yang 1, Prof. J. Zhang 2

a Henan Provincial Key Laboratory of Nanocomposite and Applications, Institute of Nanostructured Functional Materials, Huanghe Science and Technology College, Zhengzhou, Henan, 450006, P. R. China, E-mail: shourenzhang@hhstu.edu.cn; baochengyang@infm.hhstu.edu.cn.

b College of Materials Science and Engineering, Zhengzhou University, Zhengzhou 450001, P. R. China, E-mail: zjn@zzu.edu.cn

Keywords: Ru nanoclusters; N, S co-doped carbon; S-C sites; Hydrogen evolution reaction; Zinc-air battery

Experimental Section

ORR test: A CHI 760E electrochemical workstation (CH Instruments) was used to measure the electrocatalytic activities towards ORR. All the electrochemical measurements were conducted in a three-electrode configuration. The potential, measured against an Ag/AgCl electrode, was converted to the potential versus the reversible hydrogen electrode (RHE). $E_{RHE}=E_{Ag/AgCl}$ + 0.059 pH + 0.197. To prepare the working electrode, 2.5 mg each of the samples was dispersed in 336 μL isopropanol, 144 μL deionized water and 10 μL of 5 wt% Nafion aqueous solution under sonication. Then, 8 μL of this catalyst ink was pipetted onto the glassy carbon (GC) electrode (0.19625 cm^2) and dried at room temperature. The catalyst loading for the prepared catalyst on the GC electrode and commercial Pt/C (20 wt%) was 0.20 mg cm^{-2}. 0.1 M KOH/ 0.1 M $HClO_4$ aqueous solutions saturated with oxygen were employed as the electrolyte for ORR. The details for calculation of electron transferred number for ORR is given later.

The calculation of electron transferred number for ORR. On the basis of the RDE data, the electron transfer number per oxygen molecule for oxygen reduction can be determined by Koutechy-Levich equation [1-3]:

$$\frac{1}{J}=\frac{1}{J_L}+\frac{1}{J_K}=\frac{1}{B\omega^{0.5}}+\frac{1}{J_K} \quad (1)$$

$$B=0.62nFC_0(D_0)^{2/3}\nu^{-1/6} \quad (2)$$

$$J_K=nFkC_0 \quad (3)$$

Where J is the measured current density and is the electrode rotating rate (rad s^{-1}). B is determined from the slope of the Koutechy-Levichi (K-L) plot based on Levich equation (2). J_L and J_K are the diffusion- and kinetic-limiting current densities, n is the transferred electron number, F is the Faraday constant (F = 96485 C mol^{-1}), C_0 is the O_2 concentration in the electrolyte ($C_0 = 1.26 \times 10^{-6}$ mol cm^{-3}), D_0 is the diffusion coefficient of O_2 in 0.1 M KOH ($D_0 = 1.93 \times 10^{-5}$ cm^2 s^{-1}), and v is the kinetic viscosity (v = 0.01009 cm^2 s^{-1}).

For the RRDE measurements, the disk electrode was scanned cathodically at a rate of 10 mV s^{-1} and the ring potential was kept at 1.5 V versus RHE. The peroxide percentage and the electron transfer number (n) were determined by the following equations (ref) [4-5]:

$$HO_2^-\% = 200 \times \frac{I_R / N}{I_D + I_R / N} \qquad (4)$$

$$n = 4 \times \frac{I_D}{I_D + I_R / N} \qquad (5)$$

where I_d is disk current, I_r is ring current, and N is current collection efficiency of the Pt ring. N was determined to be 0.40.

***HER test**:* All the electrochemical measurements were conducted in a three-electrode configuration with an electrolyte solution of 1 M KOH/0.5 M H_2SO_4/1 M PBS, a graphite rod, and a saturated calomel electrode (SCE) reference electrode. LSV measurements were conducted in 1 M KOH/0.5 M H_2SO_4/1 M PBS with scan rate of 5 mV/s. All the potentials reported in our work were *vs*. the reversible hydrogen electrode (RHE). To prepare the working electrode, 2.5 mg each of the samples was dispersed in 225μL Isopropanol and 25 μL of 5 wt% Nafion aqueous solution under sonication. Then, 8 μL of this catalyst ink was pipetted onto the glassy carbon (GC) electrode (0.19625 cm^2) and dried at room temperature. The catalyst loading for the prepared catalyst on the GC electrode and commercial Pt/C (20 wt%) was 0.40 mg cm^{-2}.

All the linear sweep voltammetry (LSV) curves were conducted at a scan rate of 5 mV s^{-1}. The electrochemical active surface area (ECSA) of the catalysts was estimated by measuring the electrochemical double-layer capacitance (C_{dl}) using cyclic voltammetry (CV) curves under a potential window of (1 M KOH: 0.36 V-0.46V; 0.5 M H_2SO_4: 0.24 V-0.34V; 1 M PBS: 0.30 V-0.40V) *vs.* RHE at scanning rates of 20, 40, 60, 80, and 100 mV/s in the non-Faradaic potential region. The C_{dl} of various catalysts were equivalent to the linear slope, which was obtained by plotting the ΔJ/2 at different potential against the scan rate. ΔJ/2 was calculated using the equation (7):

$$\Delta j/2 = (j_{anodic} - j_{cathodic})/2 \qquad (8)$$

Then, ECSA was calculated according to the equation (9) [5-7]:

ECSA=C_{dl}/Cs (9)

Here, Cs=40 uF/cm^2, according to that the specific capacitance for a flat surface was generally found to be in the range of 20-60 μF·cm^{-2}.

Liquid Zn-air battery test: The catalyst ink recipe consists of 5.0 mg catalyst dispersed dispersed in 480 μL of DI water/isopropyl alcohol (v/v~3:7)/20 μL Nafion (5 wt.%) solution. Use 100 uL to drop onto carbon paper with a loading of 5mg cm^{-2}. And when using RuO_2 mixed with the catalyst as the working electrode of the cycle test, the ratio of 1 to 1 was added dropwise, and the loading amount was about 5 mg cm^{-2}. The air electrode was prepared by uniformly coating the as-prepared catalyst ink onto carbon paper then drying it at 60 °C for 2 h. A Zn plate was used as the anode and catalysis loaded on carbon paper are used as cathodes. Both electrodes were assembled into a home-made Zn-air battery, and 6 M KOH+0.2 M Zn $(CH_3COO)_2$/1 M NH_4Cl+0.2 M $Zn(CH_3COO)_2$ aqueous solutions was used as the electrolyte. The polarization curves were recorded by linear sweep voltammetry (5 mV s^{-1}, at room temperature) on a CHI 660D electrochemical platform. The energy density was calculated based on the applied current (I), average discharge voltage (V), service time (t), and weight of zinc consumed (w zn) as in the following equation (10) [8]:

$$Energy\ density\ (Wh\ kg^{-1}) = \frac{\mathrm{I} * \mathrm{V} * \triangle\ \mathrm{t}}{\mathrm{w}_{zn}}\ (10)$$

The specific capacity was calculated according the equation below (11):

$$Specific\ capacity\ (mAh\ g^{-1}) = \frac{\mathrm{I} * \triangle\ \mathrm{t}}{\mathrm{w}_{zn}} (11)$$

All-solid-state Zn-air battery assembly**:** A polished zinc foil (0.05 mm thickness) was used as anode. The gel polymer electrolyte was prepared as follow: polyvinyl alcohol (PVA, 5 g) was dissolved in 50 mL was added 18 M KOH (5 mL) and 2.5×10^{-3} M $Zn(CH_3OO)_2$ at 95 °C to form a homogeneous viscous solution, followed by casting on a glass disk to form a thin polymer film (thickness about 2 mm).[9] The film was then freezed in a freezer at -20 °C about 2 h, and then keep at 0 °C temperature

about 4 h. The film was thawed for 12 h before used. Then, the as-prepared catalyst film and zinc foil were placed on the two sides of PVA gel, followed by pressed Ni foam as current collector. The components were firmly pressed together by roll-pressing. No inert atmosphere or glove-box is required for the packaging.

Supplementary Figures and Tables

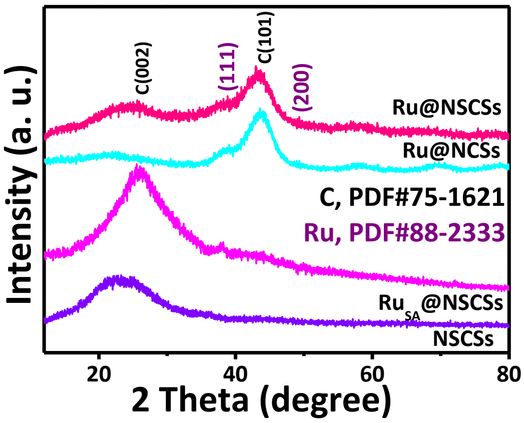

Figure S1. XRD images of Ru@NSCSs, Ru_{SA}@NSCSs, Ru@NCSs and NSCSs catalysts.

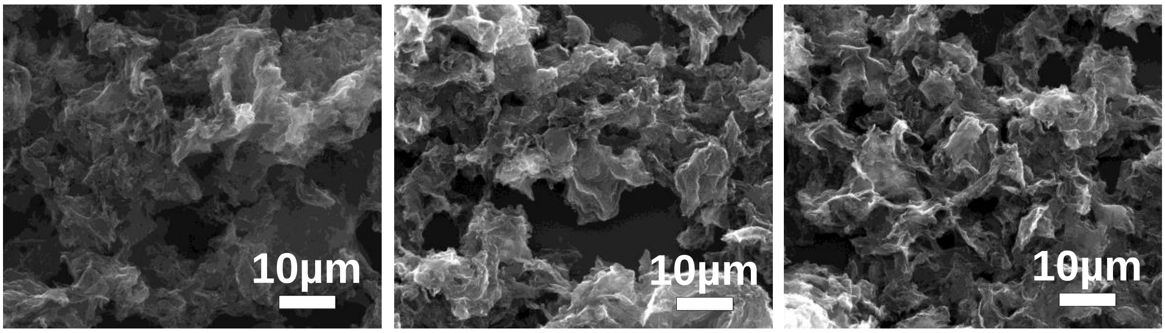

Figure S2.TEM images of NSCSs catalysts.

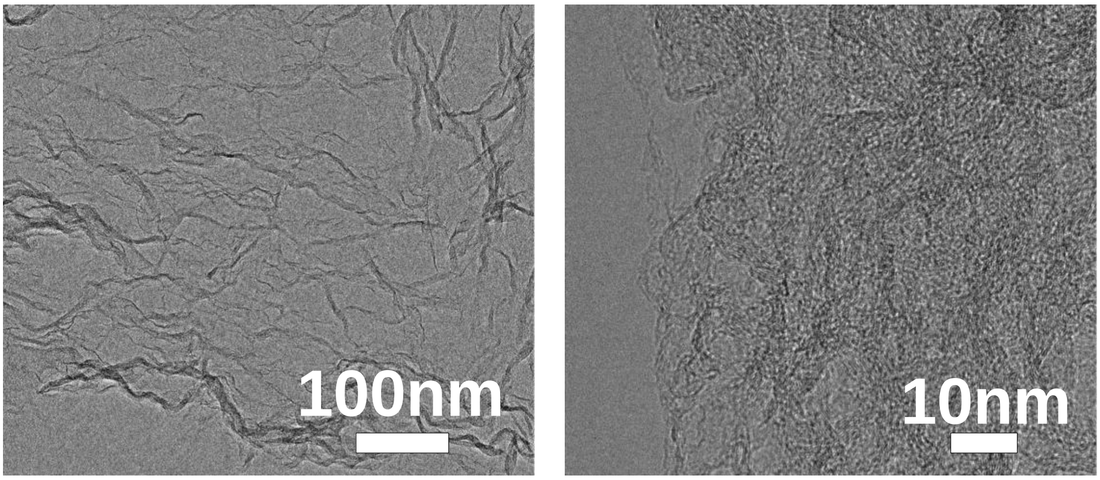

Figure S3. TEM images of the Ru_{SA}@NSCSs catalysts.

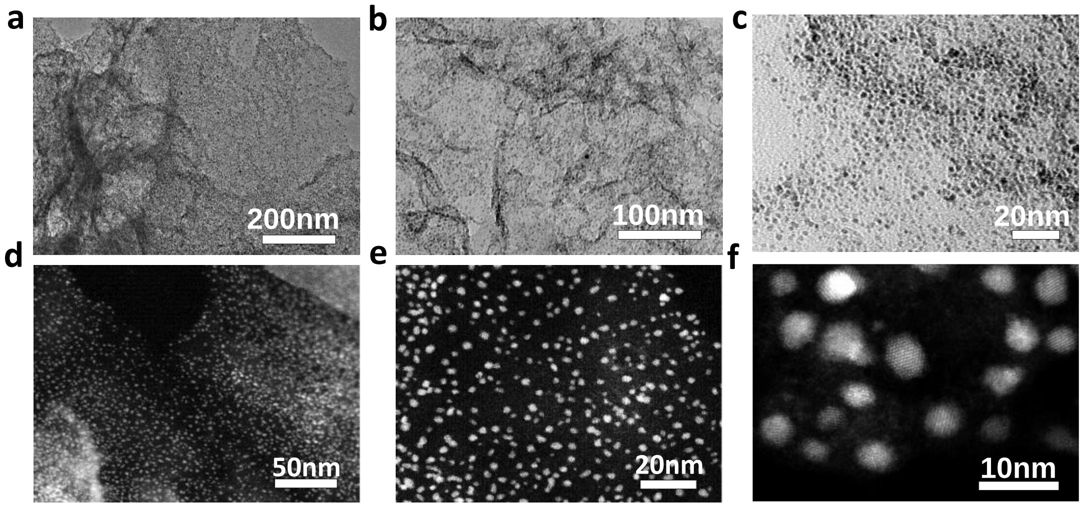

Figure S4. (a-b) TEM images and (c-f) HAADF-TEM images of Ru@NSCSs catalyst.

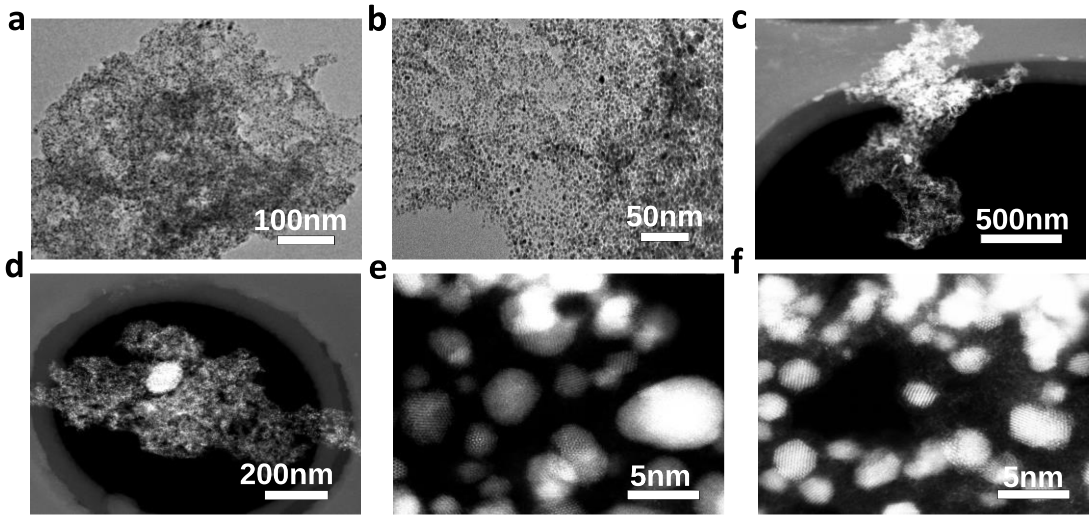

Figure S5. (a-b) TEM images and (c-f) HAADF-TEM images of Ru@NCSs catalyst.

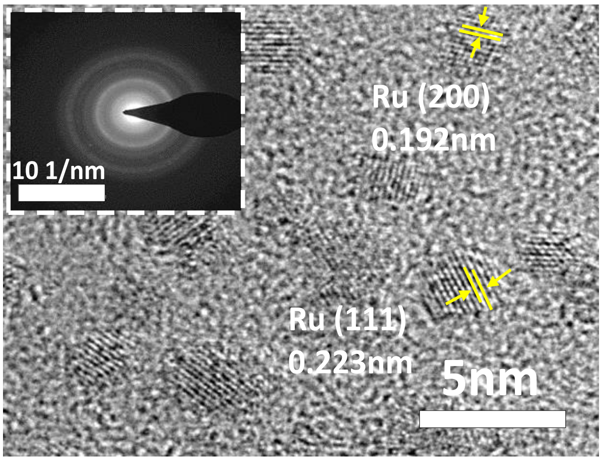

Figure S6. TEM images. Inset of the Fast-Fourier Transform (FFT) image of the composite, scale bars: 10 nm $^{-1}$.

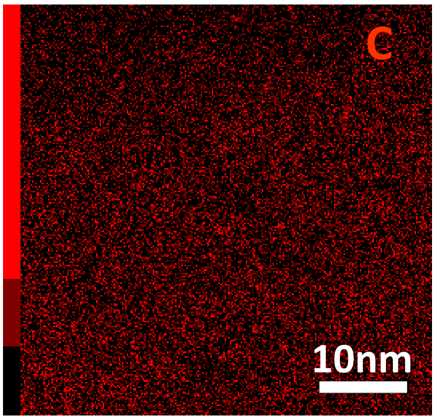

Figure S7. The EDX spectrum showing the distribution of C element.

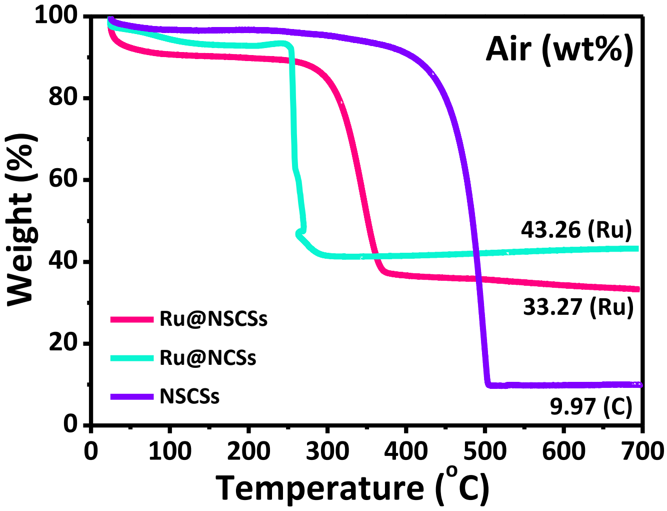

Figure S8. TGA curve of Ru@SNCSs, Ru@NCSs and NSCSs under air atmosphere at a ramping rate of 10 °C min^{-1}.

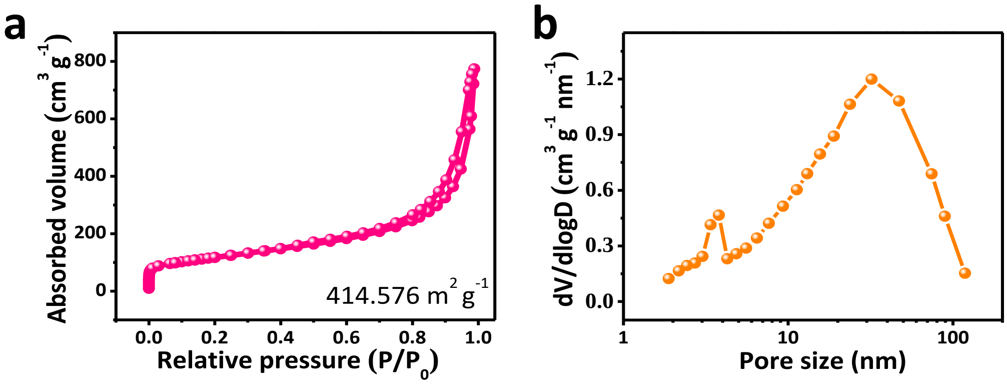

Figure S9. (a) Nitrogen (N_2) adsorption-desorption isotherm of Ru@NSCSs. (b) The inset shows the pore size distribution. The specific surface area was calculated using the BET method.

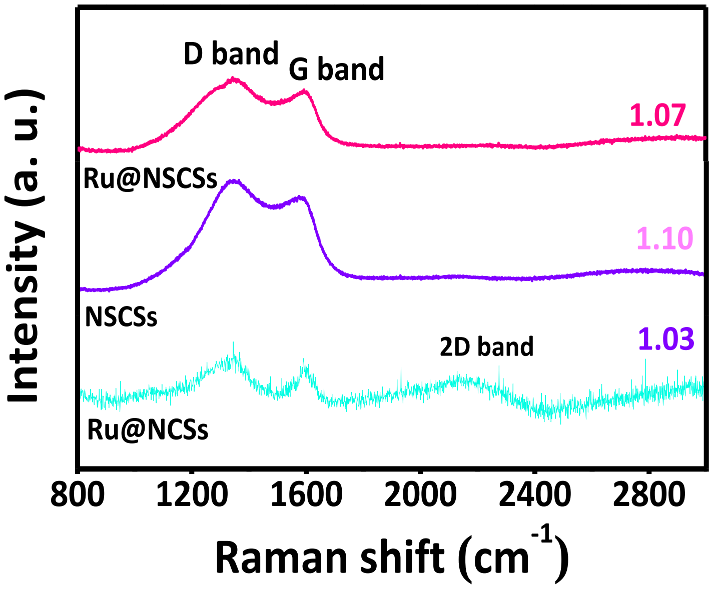

Figure S10. Raman spectra of Ru@NSCSs, Ru@NCSs and NSCSs catalysts.

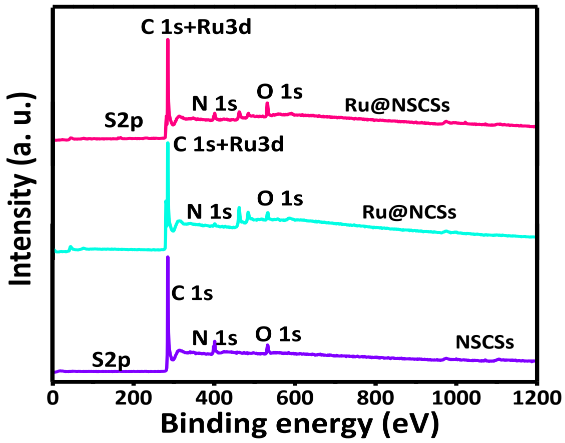

Figure S11. Survey XPS spectrum of Ru@NSCSs, Ru@NCSs and NSCSs samples.

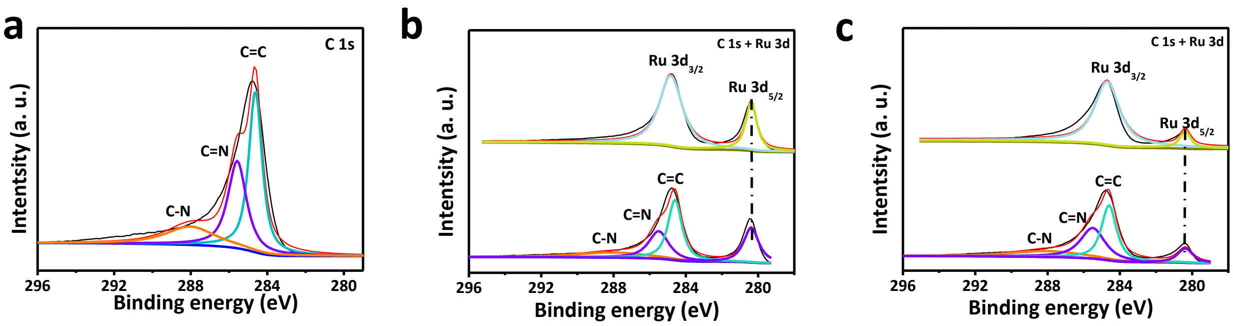

Figure S12. C 1s XPS spectra of (a) NSCSs, C 1s+Ru 3d XPS spectra of (b) Ru@NCSs and (c) Ru@NSCSs samples.

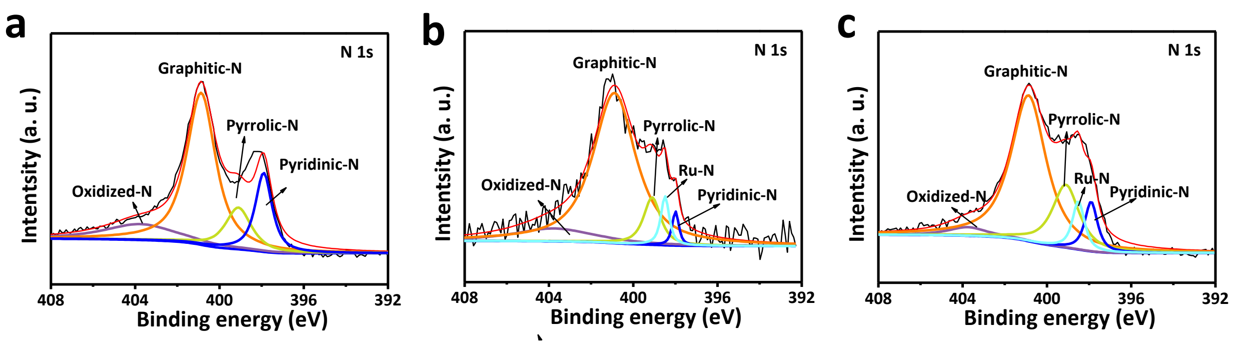

Figure S13. N 1s XPS spectra of (a) NSCSs, (b) Ru@NCSs and (c) Ru@NSCSs samples.

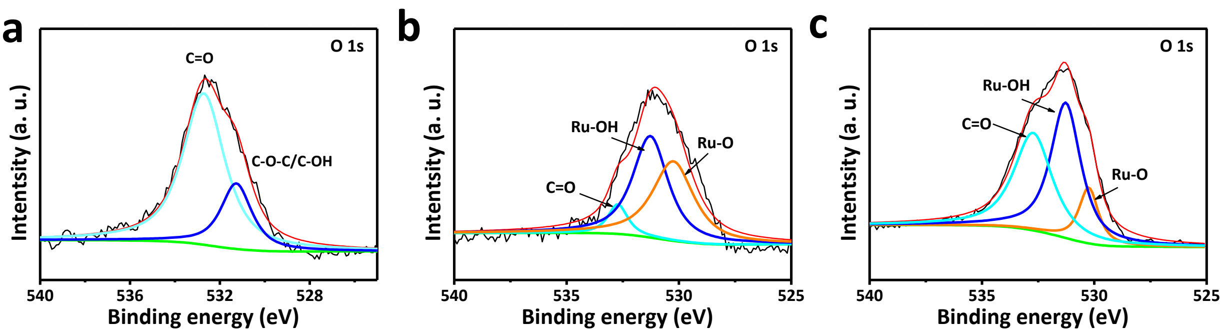

Figure S14. O 1s XPS spectra of NSCSs, Ru@NCSs and Ru@NSCSs catalysts.

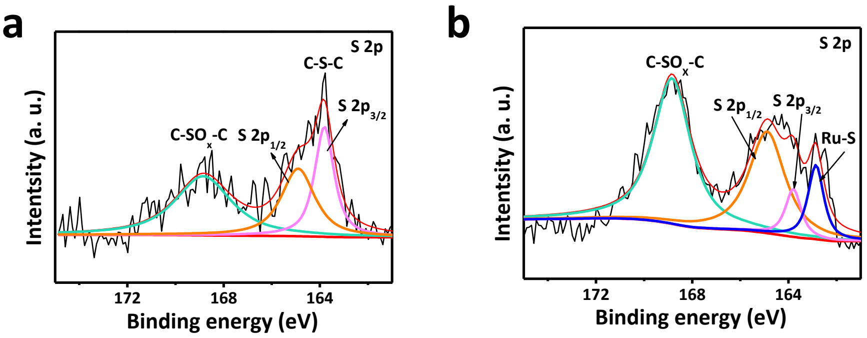

Figure S15. S 2p XPS spectra of (a) NSCSs and (b) Ru@NSCSs samples.

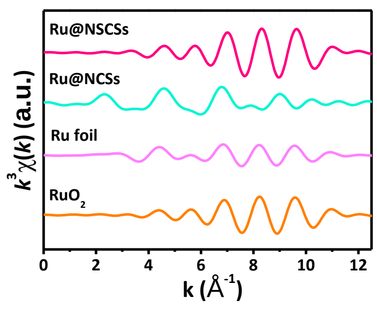

Figure S16. EXAFS of Ru@NSCSs and Ru@NCSs, RuO_2 and Ru foil in k-space.

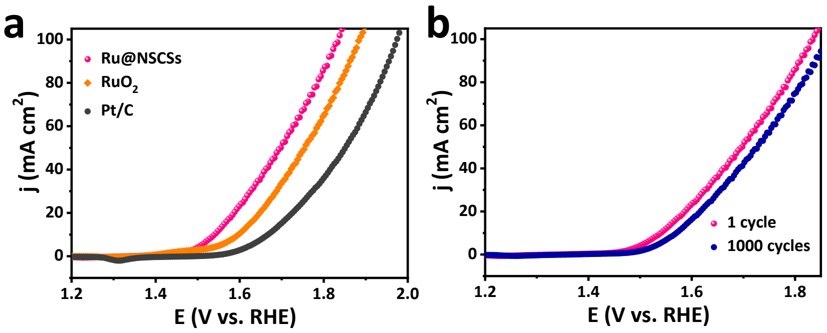

Figure S17. The OER polarization curves of Ru@NSCSs, RuO_2, and commercial Pt/C catalyst at a sweep rate of 5 mV s^{-1} in 1 M KOH solution. (b) The polarization curves were recorded initially and after 1000 cycles at a sweep rate of 100 mV s^{-1}.

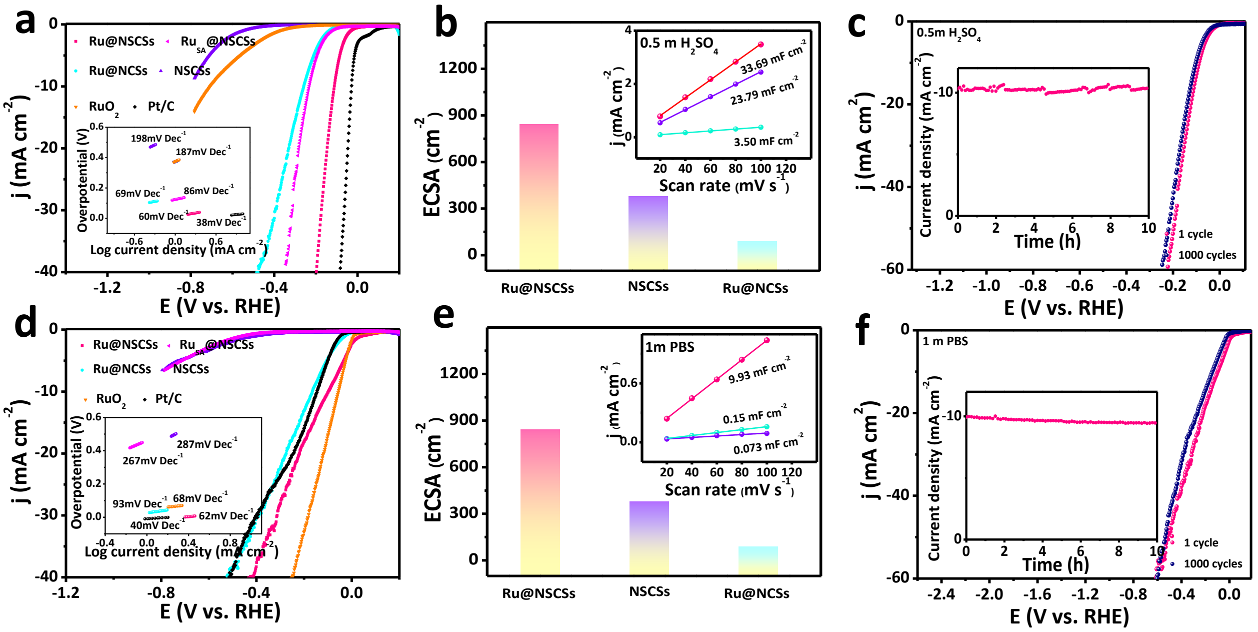

Figure S18. HER polarization curves of Ru@NSCSs, Ru_{SA}@NSCSs, Ru@NCSs, NSCSs, RuO_2 and commercial Pt/C catalyst at a sweep rate of 5 mV s^{-1} in (a) 0.5 M H_2SO_4 solution, (d) 1 M PBS solution. Inset correspond to the Tafel plots for these catalysts. (b, e) ECSA values of Ru@NSCSs, Ru@NCSs, and NSCSs catalysts. Inset shows estimation of C_{dl} by plotting the half of current density variation ($\Delta j = j_{anodic} - j_{cathodic}$). ECSA values at the overpotentials of 200 mV. (c, f) The polarization curves were recorded initially and after 1000 cycles. Inset shows the chronopotentiometry of Ru@NSCSs catalysts.

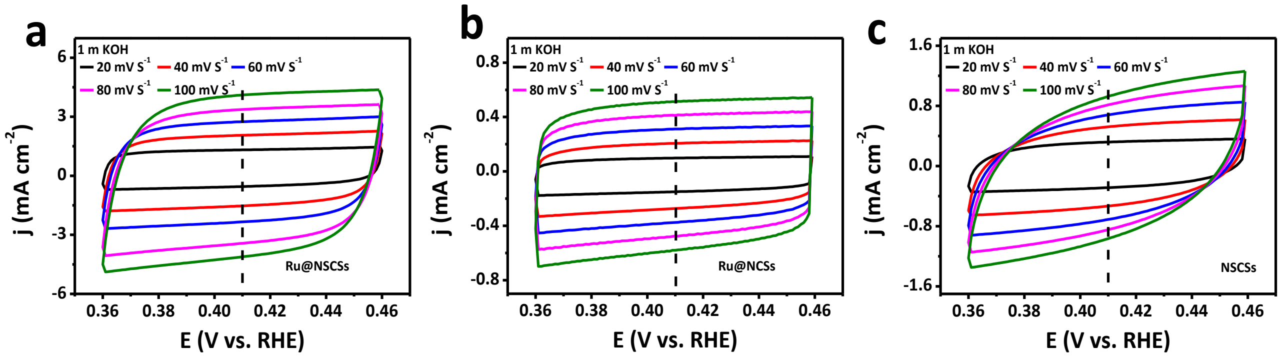

Figure S19. CV curves of (a) Ru@NSCSs, (b) Ru@NCSs, and (c) NSCSs at different scan rate of 20 mV S^{-1} to 100 mV S^{-1} in 1 M KOH solution.

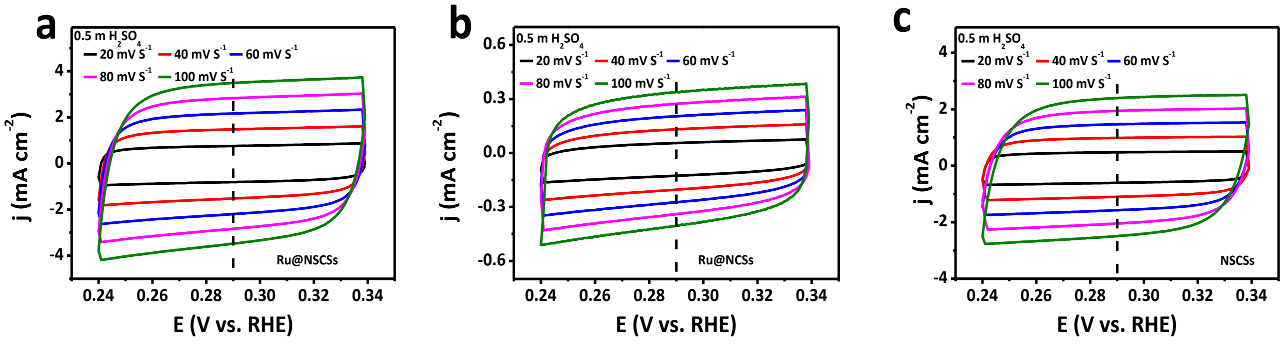

Figure S20.CV curves of (a) Ru@NSCSs, (b) Ru@NCSs, and (c) NSCSs at different scan rate of 20 mV S^{-1} to 100 mV S^{-1} in 0.5 M H_2SO_4 solution.

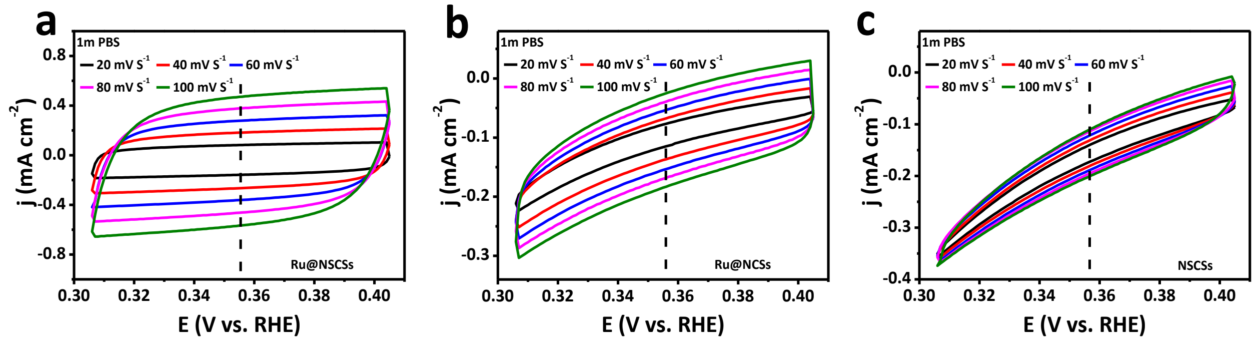

Figure S21.CV curves of (a) Ru@NSCSs, (b) Ru@NCSs, and (c) NSCSs at different scan rate of 20 mV S^{-1} to 100 mV S^{-1} in 1 M PBS solution.

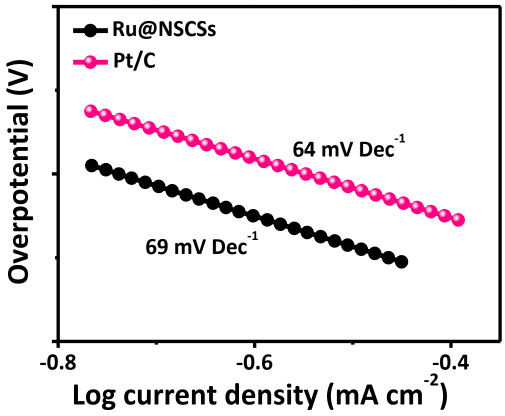

Figure S22. The corresponding Tafel plots of Ru@NSCSs, Pt/C catalysts

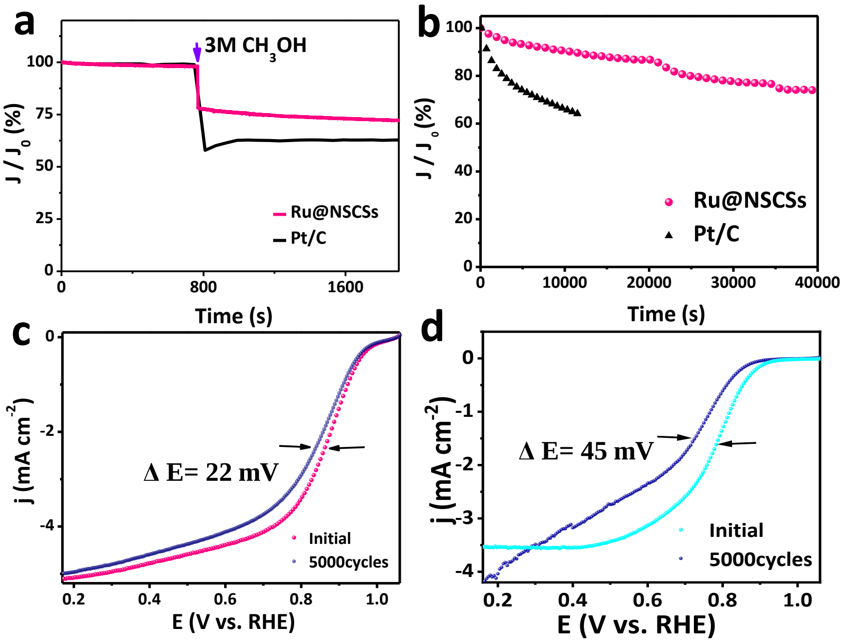

Figure S23. (a) Chronoamperometric response for Ru@NSCSs and Pt/C electrode at 0.75 V (*vs.* RHE) after the introduction of 9.7 mL of CH_3OH into 230.3 mL of 0.1 M KOH solution. (b) Chronoamperometry response of Ru@NSCSs and Pt/C catalyst 0.75 V (vs. RHE). (c-d) The Ru@NSCSs and Ru@NCSs of ORR polarization curves (1600 rpm) before and after 5,000 cycles.

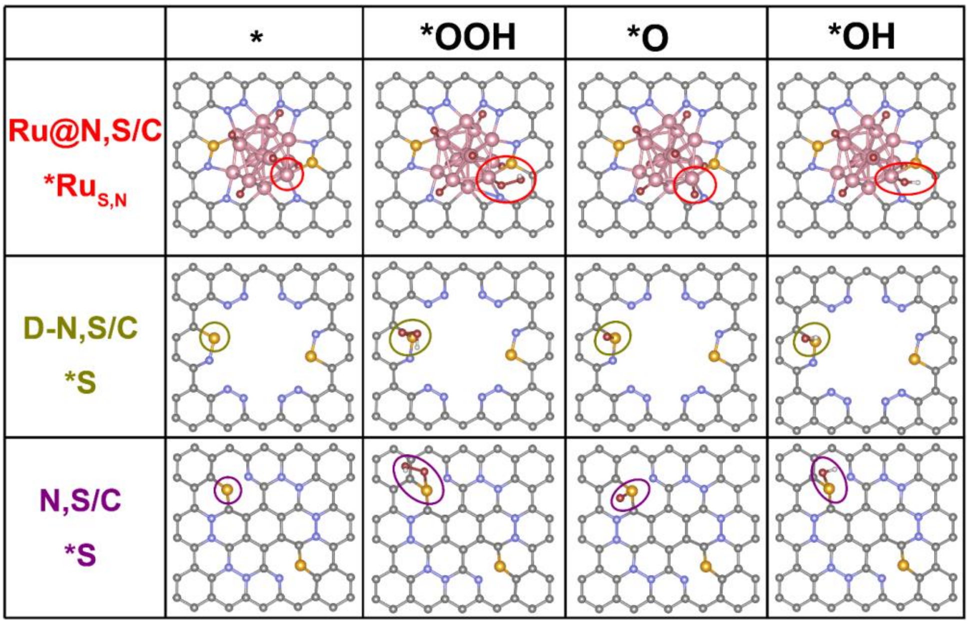

Figure S24. The configurations of intermediate of OOH*, O* and OH* on different active sites of S atom in N, S/C and D-N, S/C and Ru in Ru@N, S/C. The white, grey, blue, yellow, red, and pink balls represent H, C, N, S, O, and Ru atoms, respectively.

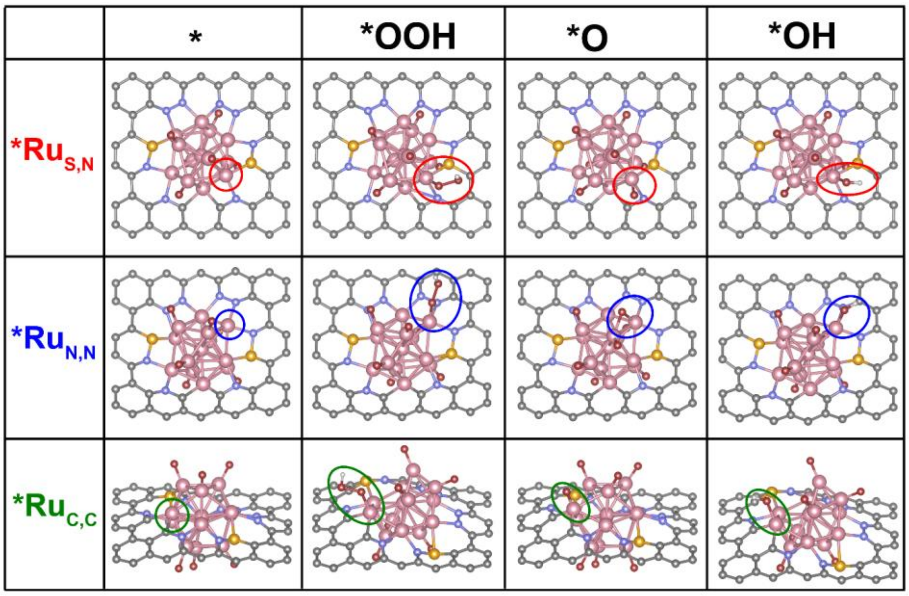

Figure S25. The configurations of intermediate of OOH*, O* and OH* on different active sites of *Ru$_{S,N}$, *Ru$_{N,N}$, and *Ru$_{C,C}$ in Ru@N, S/C. The white, grey, blue, yellow, red, and pink balls represent H, C, N, S, O, and Ru atoms, respectively.

Tables

Table S1. The content of metals in different catalysts from ICP.

Sample Name	The content of metal from ICP (wt%)
**Ru@NSCSs**	**2.66**
**Ru@NCSs**	**2.72**

Table S2. XPS results analysis for the prepared samples. (at %)

Sample Name	C (Atom %)	O (Atom %)	N (Atom %)	S (Atom %)	Ru (Atom %)
**Ru@NSCSs**	**80.17**	**7.31**	**6.29**	**0.85**	**5.38**
**Ru@NCSs**	**87.47**	**3.89**	**2.23**	**~**	**6.41**
**NSCSs**	**86.35**	**4.2**	**9.2**	**0.25**	**~**

Table S3. Surface and pore related parameters from N_2 adsorption isotherms of samples.

Sample Name	BET surface area (m^2 g^{-1})	Total pore volume (cm^3 g^{-1})	Micropore volume (cm3 g-1)	Pore size (nm)
**Ru@NSCSs**	414.576			3.8/32.11

Table S4. Comparison sample of the as-prepared catalysts electrocatalysts for the hydrogen evolution reaction.

Catalyst	electrolyte	η =10 (10 mA cm^{-2})
**Ru@NSCSs**	*1M KOH*	*5*
	0.1M H_2SO_4	*89*
	1M PBS	*98*
**Ru@NCSs**	*1M KOH*	*198*
	0.1M H_2SO_4	*258*
	1M PBS	*163*
**NCSs**	*1M KOH*	*172*
	0.1M H_2SO_4	~
	1M PBS	~
**RuO_2**	*1M KOH*	*154*
	0.1M H_2SO_4	*708*
	1M PBS	*147*
**Pt/C**	*1M KOH*	*38*
	0.1M H_2SO_4	*29*
	1M PBS	*56*

Table S5. Recent advances in ruthenium-based electrocatalysts for the hydrogen evolution reaction.

Catalyst	electrolyte	*η =10* (10 mA cm^{-2})	Reference
**Ru@NSCSs**	*1M KOH*	*5*	***This work***
	0.1M H_2SO_4	*89*	
	1M PBS	*98*	
**Ru_2B_3@BNC**	*1M KOH*	*7*	*Nano energy*, **2020,** 75, 104881
	0.1M H_2SO_4	*41*	
	1M PBS	*58*	
**RuP_2@NPC**	*1M KOH*	*52*	*Angew. Chem. Int. Ed.*, **2017**, *56*, 11559
	0.1M H_2SO_4	*38*	
	1M PBS	*57*	
**$NiRu_{0.13}$-BDC**	*1M KOH*	*34*	*Nat Commun* **2021**, *12,* 1369
	1M HCl	*13*	
	1M PBS	*36*	
**RuM/CQD**	*1M KOH*	*13*	*Angew. Chem. Int. Ed.*, **2020**, *59*, 1718
	0.1M H_2SO_4	*58*	
	1M PBS	*18*	
**Ru@WNO-C**	*1M KOH*	*24*	*Nano Energy*, **2021**, *80*, 105531
	0.1M H_2SO_4	*172*	
	1M Na_2SO_4	*358*	
**Ru/C-H_2O/ CH_3CH_2OH**	*1M KOH*	*53*	*Applied Catalysis B: Environmental,* **2019**, *258*, 117952
	0.1M H_2SO_4	*35*	
	3.5wt.%NaCl	*93*	
**RuSA-N-$Ti_3C_2T_x$**	*1M KOH*	*27*	*J. Mater. Chem. A,* **2020**, *8*, 24710
	0.1M H_2SO_4	*23*	
	1M PBS	*81*	
**Ru@CN-0.16**	*1M KOH*	*32*	*Energy Environ. Sci.,* **2018**, *11*, 800
	0.1M H_2SO_4	*126*	
	1M PBS	*100*	

Table S6. Comparison sample of the as-prepared catalysts ORR electrocatalysts in O_2 saturated 0.1 M KOH solution.

Catalyst	$E_{1/2}$ (V *vs*. RHE)	J_k (V *vs*. RHE)
**Ru@NSCSs**	**0.854**	**5.115**
**Ru$_{SA}$@NSCSs**	**0.79**	**4.12**
**Ru@NCSs**	**0.652**	**4.178**
**NSCSs**	**0.831**	**4.946**
**Pt/C**	**0.846**	**5.126**

Computational Section

All the DFT calculations were carried out with the projector augmented wave (PAW) [10] method as implemented by Vienna ab initio simulation package (VASP). [11] The exchange-correlations were treated by the generalized gradient approach (GGA) in the form of Perdew-Burke-Ernzerhof (PBE) functional.[12] The energy cutoff of the wave function was set as 400 eV. Considering the unit cell is so huge that the Brillouin zone was sampled by the Γ point. [13-14] All the atoms were allowed to fully relax until the convergence tolerance of energy and maximum force were smaller than 10^{-5} eV/atom and 0.05 eV/Å, respectively. The vacuum spacing of 20 Å was set to avoid the interaction between two periodic units. The S and N doped carbon nanosheet (N, S/C) was modeled by replacing twelve C atoms with ten N atoms and two S atoms in the 8 × 8 graphene sheet. Then the inner twelve C atoms encircled by the N and S atoms were removed from the N, S/C, forming the defective S and N doped carbon nanosheet (D-N, S/C) to capture ruthenium nanocluster subsequently. We adopt an icosahedral Ru_{13} as the simplified model of the ruthenium cluster, in which partially ruthenium atoms were oxidized during the oxygen reduction reaction. [14] Finally, the ruthenium nanocluster with distorted icosahedral was embedded on the hole of D-N, S/C, coordinating with surrounding S, N and C atoms (Ru@N,S/C). Obviously, there are three different type Ru active sites, i.e., $Ru_{S,N}$ (one Ru atom coordinating with one S and N atom), $Ru_{N,N}$ (one Ru atom coordinating with two N atom), and $Ru_{C,C}$ (one Ru atom coordinating with two C atom).

We employed the computational hydrogen electrode (CHE) method developed by Nørskov et al. [15] to compute the free energies of all elementary steps. In the first step of four-electron pathway, an O_2 molecule reacts with a proton-electron pair producing *OOH. Next, the *OOH is protonated to form *OH and release the first H_2O, then the *O is further protonated to form *OH, and finally the *OH will be protonated to form the second product H_2O, recovering the active site for next round of ORR. In detail the elementary steps as follows [16]:

$$H^{+} + e^{-} + O_2 + * \rightarrow *OOH \qquad (1)$$

$$H^{+} + e^{-} + {*OOH} \rightarrow {*O} + H_2O \qquad (2)$$

$$H^{+} + e^{-} + {*O} \rightarrow {*OH} \qquad (3)$$

$$H^{+} + e^{-} + {*OH} \rightarrow {*} + H_2O \qquad (4)$$

Where the symbol "*" represents the active site, and the "*OOH", "*O", "*OH" represent the intermediate species adsorbed on the active sites. For each step, the reaction free energy (ΔG_{ORR}) is calculated by

$$\Delta G_{\mathrm{ORR}} = \Delta E + \Delta ZPE - T\Delta S$$

where, ΔE, ΔZPE, and ΔS are the difference in total energy, zero-point energy, and entropy between final and initial states, respectively, T is the temperature at 298.15 K. Zero-point energies for the ORR intermediates were obtained from the vibrational frequencies after structural optimization, and those of free gas-phase molecules were obtained from NIST database.[17] Since oxygen molecule has a complicated electronic structure which cannot be described accurately by DFT, the free energy change of the total reaction $H_2O \rightarrow 1/2O_2 + H_2$ is fixed in this work to avoid the direct calculation of O_2 molecule. The free energy for $H_2O \rightarrow 1/2O_2 + H_2$ is found to be 2.46 eV. Hence $G_{O_2} = 4.92 - 2E_{H_2} + 2E_{H_2O} - (\Delta ZPE - T\Delta S)_{2H_2O \rightarrow O_2 + 2H_2}$. The overpotential η is calculated with the formula of $\eta = U_{eq} + U$, where U_{eq} is the equilibrium potential of 1.23 V for ORR, and U = ΔG_{max} / e, where ΔG_{max} is maximum Gibbs free energy change among the four elementary steps during the ORR process. [18]

Reference

[1] Y. Y. Guo, P. F. Yuan, J. Zhang, Y. Hu, Y.; I. S. Amiinu, X. Wang, J. Zhou, H. Xia, Z. Song, Q. Xu, S. Mu. *ACS Nano* **2018**, *12*, 1894-1901.
[2] I. S. Amiinu, Z. H. Pu, X. B. Liu, K. A. Owusu, H. G. R. Monestel, F. O. Boakye, H. N. Zhang, S. C. Mu. *Adv. Funct. Mater.* **2017**, *27*, 1702300.
[3] X. X. Yang, M. Y. Wang, M. J. Zachman, H. Zhou,Y. H. He, S. W. Liu, H. Y. Zang, Z. X. Feng, G. Wu. *Small Sci*. *2021*, *1*, 2100046.
[4] Y. Zheng, H. Song, S. Chen, X. H. Yu, J. X. Zhu, J. S. Xu, K. A. I. Zhang, C. Zhang, T. X. Liu. *Small* **2020**, *16*, 2004342.
[5] S. J. Peng, H. J. Huang, D. S. Yu, F. Hu, S. C. Huang, J. N. Song, H. Y. Chen, L. L. Li. *Angew. Chem.Int. Ed*. **2022**, *61*, e2021160.
[6] X. Q. Mu, J. N.Gu, F. Y. Feng, Z. Y. Xiao, C. Y. Chen, S. L. Liu, S. C. Mu. *Adv. Sci*. **2020**, 2002341.
[7] H. B. Zhang, W. Zhou, X. F. Lu, T. Chen, X. W. Lou. *Adv. Energy Mater*, **2020**, *10*, 2000882.
[8] Y. Y. Qiao, P. F. Yuan, Y. F. Hu, J. N. Zhang, S. C. Mu, J. H. Zhou, H. Li, H. C. Xia, J. He, Q. Xu. *Adv. Mater*. **2018**, *30*, 1804504.
[9] Y. Y. Guo, P. F. Yuan, J. N. Zhang, H. C. Xia, F. Y. Cheng, M. F. Zhou, J. Li, Y. Y. Qiao, S. C. Mu, Q. Xu. *Adv. Funct. Mater.* **2018**, *28*, 1805641.
[10] P. E. Blöchl. *Phys. Rev. B* **1994**, *50*, 17953-17979.
[11] G. Kresse, J. Furthmüller. *Phys. Rev. B* **1996**, *54*, 11169-11186.
[12] J. P. Perdew, K. Burke, M. Ernzerhof. *Phys. Rev. Lett*. **1996**, 77, 3865-3868.
[13] H. J. Monkhorst, J. D. Pack. *Phys. Rev. B* **1976**, *13*, 5188-5192.
[14] Q. Wang, C.-Q. Xu, W. Liu, S.-F. Hung, H. B. Yang, J. Gao, W. Cai, H. M. Chen, J. Li, B. Liu. *Nat. Commun*. **2020**, *11*, 4246.
[15] J. K. Nørskov, J. Rossmeisl, A. Logadottir, L. Lindqvist, J. R. Kitchin, T. Bligaard, H. Jonsson. *J. Phys. Chem. B* **2004**, *108*, 17886-17892.
[16] Q. Lv, W. Y. Si, J. J. He, L. Sun, C. F. Zhang, N. Wang, Z. Yang, X. D. Li, X. Wang, W. Q. Deng, Y. Z. Long, C. S. Huang, Y. L. Li. *Nat. Commun*. **2018**, *9*, 3376.
[17] http://webbook.nist.gov/chemistry/.
[18] Y. R. Ying, K. Fan, X. Luo, J. L. Qiao, H. T. Huang. *J. Mater. Chem. A*, **2021**, *9*, 16860-16867.
